# Aphasia in Brain Disease

**Published:** 1885-09

**Authors:** Mark H. O’Daniel

**Affiliations:** Assistant Physician Georgia State Lunatic Asylum, Milledgeville, Georgia


THE ATLANTA MEDICAL AND SURGICAL JOURNAL.

VOL. II. SEPTEMBER, 1885. NO. 7.

### Original Communications.

### APHASIA IN BRAIN DISEASE.

PRESENTED TO THE MEDICAL ASSOCIATION OF GEORGIA AT ITS THIRTY-SIXTH ANNUAL SESSION, IN SAVANNAH, APRIL 15TH, 1885, BY MARK H. O'DANIEL, M. D., ASSISTANT PHYSICIAN GEORGIA STATE LUNATIC ASYLUM, MILLEDGEVILLE, GEORGIA.

By the term Aphasia, we mean a condition in which a patient can think and can understand, but has no power to render his thoughts into articulate speech, and which is of cerebral origin. While the malady is not of very rare occurrence, still it is one not frequently met with, and I think with the forms, with which it is accompanied, quite uncommon. I have recently read the reports of quite a number of interesting cases, but in no instance have I been able to note one similating this.

On the 13th of September last, Dr. I. was received in this institution as an insane patient, parties accompanying him giving the following history: South Carolinian by birth; age fifty years; widower; duration of trouble only a few days; cause supposed to have been remote pre-disposing tendency to neurosis, together with a combination of exciting causes, viz.: Domestic trouble, with whisky and opium habit—both suicidal and homicidal—sl[illegible] irregularly, eats very well, general health very good. The pa-tient was assigned a ward and strict instructions given the attendant relative to his suicidal and homicidal tendency. When visited he was found quiet and orderly, but in a rather depressed and stupid state, which we thought due to more decided doses of morphia than usual, and again, to the mortification and embarrassment which he was probably undergoing at the time, on account of having been committed without his knowledge or consent, he being impressed with the idea that he was being brought merely for a consultation. Upon questioning him the fact was at once revealed, that he was unable to give utterance to his ideas, only in a partial and very imperfect manner. There was found no paralysis about the tongue or lips to account for this. The act of deglutition was normal, and without the slightest difficulty performed. He was found especially at a loss to call proper names, being unable to speak his own, the names of persons who accompanied him, the name of his own county, etc. While he had control of language somewhat, knowing him from his history, addicted to the whisky and opium habit, he was next asked in regard to it and his daily consumption. With what language he had control, and by means of signs and gestures, which he had quickly learned to make use of, he could make himself pretty well understood on a good many subjects. Not being able to say morphine and number of grains, whisky and number of ounces, he representing his fingers as grains would give the amount of morphia, and showed to us the size bottle, thus giving the quantity of whisky. He could neither say brandy, beer, or wine, but could say liquor. He would say always, Doctor, I know full well what I want to say, if I could only speak it, and would point to his head as the seat of his trouble, and state that he thought all would soon come back. He seemed to realize fully his condition, and was generally bright and hopeful. He would often feel that he could call his name, or that of some officer or patient, and would rush to them in order that they might hear him. He would halt as if in profound astonishment, and say, "I had the word but have lost it; can't call it now to save my life;" says yes for no, and *vice versa*, knowing at the same time they are wrong, confuses syllables in words, appears much worried and angry that he cannot express himself. I was sitting with him in my office a short time ago, during which time he was busily engaged preparing a walking cane for a friend. He had a good deal to say about the cane—its beauty, the kind of wood, the place from which he obtained it, etc. I asked him the name of his friend to whom he intended presenting it. His reply was: "I of course know, but can't call his name—will point him out to you," which he did, the person being an officer of the institution; and I will state right here, that up to a very short time ago, he was unable to call by name a single person here or elsewhere.

After he had finished scraping the roughness from the cane, he asked for something with which he could polish it. I inquired what he desired, and his answer was: "I can't tell you, but if you will be kind enough to walk around (pointing to the apothecary, which he could not call), I will point out to you." I complied with his request, and so soon as we entered, he, with a bright smile upon his face, pointed out the desired ingredients.

One remarkable feature in his case, and I have noted similar occurrences in the reports of like cases, is his power over the use of profane language. He is quite passionate, and when aroused, or becomes angry, ofttimes on account of his inability to say what he wishes, or thinks of being deceived when brought here, becomes quite fluent in the usage of words of profane kind, seemingly having complete control of the entire vocabulary. His recollection and memory of words is perfectly in tact. He is a fine violinist, and has often times delighted us with his sweet strains of music. He understands perfectly anything you may say or direct to him, and can follow up a train of thoughts consecutively, when spoken or read aloud to him, the import of words and sounds being perfect, but the power of transmission on his part at fault, showing evidently that the first part of the path, through which the motor stimulus would have to pass, in order to incite the necessary combined muscular movement is broken up or damaged.

Dr. Bastian says of these cases: "We presume that the afferent fibres connecting the auditory perceptive centers of the cerebral hemispheres, and also these latter centers themselves, are in tact, so that the spoken sounds revive their accustomed impressions in the hemispheres, these being perceived as words symbolic of things, and ideas, which being duly appreciated by the individual, as they are conjoined up, suggest to him thoughts which they are intended to convey."

The diagnosis made here was partial aphemia.

Typical cases of aphemia are very rare, where patients recovering from unconsciousness are found speechless and remain so for weeks and months, having in tact other faculties. In this instance, the aphemia is not complete, besides, is accompanied with the agraphic variety, agraphia.

By this term, we mean an inability on part of the patient to write or read writing. While I cannot say it is complete in this case, yet it is more decided than the former. I have seen the patient frequently when he was totally incapable of reading or writing a single word, which was more generally the rule than the exception. I have on several occasions tested him by means of a newspaper, or by writing some name with which I knew him to be familiar, when he would say to me, "the whole is a complete blank to-day—can't read it." Then again, I have seen him perhaps on the following day, when he could read them understandingly, giving me unmistakable evidence of his comprehension by telling me that he had frequently visited the city or town in which the paper was published, or that the person named was a dear friend or relative, which I knew to be the case. During the past few weeks he has been able a few times to write his name, and once a short note, which I failed to see. In writing, he is as likely to commence with the middle or last letter of a word as the first, knowing it to be wrong, but by repeated effort could overcome it, and oftentimes fail and give up in disgust. I have been endeavoring to get a letter from him, which would show more fully his condition than I can picture, but have failed to do so.

He is a most eloquent gentleman—a man who has been blessed with the best educational advantages; highly cultured, polished, and possessed with the keenest sense of every propriety. He has always been quiet and orderly, and has never exhibited the slightest desire to do violence, either to himself or other parties. The suicidal and homicidal part of his history, I think, can be safely attributed to times when under the influence of whisky, prior to this trouble, he having been a drinker, often to excess, for a number of years, and bearing the reputation of a man of passionate nature and strong resolution. There has never been the slightest insanity noticeable since his admission, though there is often in these cases more or less mental disturbance.

*Pathological Evidence.*—The skin over the region of the head, moist and of natural temperature; pulse and respiration normal; other organs performing their natural functions. He complains of severe pains in the region of the left frontal lobe, extending frequently across the forehead, which is more severe at times. He complained later on of pain in his back and lower extremities, together with a feeling of general sensitiveness. This pain and general hyperæsthetic condition is attributable, I think, to the reduction in his whisky and opium. An increase would relieve him. I have had opportunity to notice numbers of opium cases, and yet have my first to see who did complain of like symptoms, as the opium was gradually withdrawn.

The lesion is, as given by best writers upon the subject, found to exist in the lower portion of the third left frontal convolution more frequently, produced either by a clot, embolus, softening tumor or traumatic influence, and here I will mention, I learned from the patient that a good many years ago he was thrown from a buggy, receiving an injury just at the occipito parietal junction, but that it has never caused him the slightest pain or trouble. A small depression, however, was found. Whether or not there can be a supervening trouble that has caused, at this late day, the existing malady, I am unable to say, though I have every reason to believe to the contrary. In my opinion the most plausible cause was the rupture of an arterial branch, which resulted in the formation of a clot.

I was informed that the patient had been endeavoring to reduce his morphine, and had been feeling quite unwell for several days, when a party of friends came by his office and insisted that he should go hunting with them. He declined, stating that he felt too unwell, but they prevailed, and he gratified their request, after taking his morphine for the day, being accustomed to one dose only. While out, he suddenly had a paroxysm, in which he became unconscious. He was carried home, and remained in this condition for four days. When consciousness re-appeared he was found in this aphasic state. The paroxysm was evidently a convulsion. In a conversation with the patient, I obtained a part of this evidence from him. In this condition testimony was taken under a writ *lunatico inquirendo*, and he was adjudged and sent to the asylum.

*Prognosis.*—While there has been marked improvement in his condition, we entertain grave doubt as to his recovery. He is by no means safe, for the fact that there may occur at any time a second hemorrhage, which in all probability will prove fatal. I am of the opinion that such a result will be his fate.

The treatment is very simple. Our first means was to reduce cautiously the whisky and opium. Especially the former, the patient always appearing a great deal worse under its influence. As he seemed to have what might be termed his good and bad days, so to speak, we tried what virtue there might be in anti-periodics, and placed him upon alteratives—most prominently among which was iodide of potassium. Relieved him of pain in his head and caused him to sleep at night, as much as possible, by free administration of the bromides, but often had to resort to increased doses of opium, which only seemed to afford relief. His general health and comfort was cared carefully for, and his surroundings and associations made as pleasant as our means and ability rendered. I have reported the case, not that I felt I could offer anything new upon the subject, as many of the best authors have written most ably and exhaustively, but on account of its rarity.

NOTE—The writer wishes to express his indebtedness to Drs. Powell and Hall for the case.

